# Case Report: Pediatric Thyroid Storm Presenting to the Emergency Department with Afebrile Seizure

**DOI:** 10.5811/cpcem.1359

**Published:** 2024-01-23

**Authors:** Daniel J. Klein, Emily Rose

**Affiliations:** Los Angeles General Medical Center, Keck School of Medicine of the University of Southern California, Department of Emergency Medicine, Los Angeles, California

**Keywords:** *thyroid storm*, *pediatric afebrile seizure*, *thyrotoxicosis*, *emergency medicine*, *case report*

## Abstract

**Introduction:**

Seizures are a common presenting complaint and account for approximately 1% of total emergency department (ED) visits. Seizures are especially common in children less than five years old as they have a lower seizure threshold as compared to adults. One potentially dangerous etiology that is far less common, especially in children, is thyroid storm, the extreme manifestation of hyperthyroidism.

**Case Report:**

We describe the case of a 3-year-old girl who presented to the ED with an afebrile seizure but was found to be in thyroid storm. This case should serve as a reminder for emergency physicians to consider thyroid disease when evaluating patients presenting with seizures.

**Conclusion:**

Although most pediatric seizures are self-limited and frequently benign, it is imperative that the emergency physician evaluate for and rule out any potentially associated dangerous conditions such as thyroid storm.

Population Health Research Capsule What do we already know about this clinical entity?

*Thyroid storm is a potentially life-threatening condition that can cause seizures as well as multiorgan failure.*

What makes this presentation of disease reportable?

*Seizures are common in young children, but thyroid storm/thyrotoxicosis as an etiology is not.*

What is the major learning point?

*Thyroid storm should be considered for any patient with a new-onset afebrile seizure, especially those with exophthalmos, a palpable goiter, and family history.*

How might this improve emergency medicine practice?

*By increasing awareness of this rare condition clinicians will be more able to diagnose and treat it before irreversible organ dysfunction occurs.*

## INTRODUCTION

Seizures are a common presenting complaint and account for approximately 1% of total emergency department (ED) visits^1^; they are common in young children.^2^ While febrile seizures are more common, affecting 2–5% of children, approximately 1% of children <14 years in age have had one afebrile seizure.^2^ ^,^ ^3^ The differential diagnosis of a first-time afebrile seizure is broad, including both benign and life-threatening etiologies.^4^ One less common but potentially life-threatening cause of seizures is thyroid storm. Thyroid storm is the extreme manifestation of hyperthyroidism and brings with it a mortality rate between 7–30%.^2^ ^,^ ^5^ ^–^ ^7^ As such, it is an important consideration in patients of all ages presenting to the ED with seizures, but especially important for pediatric patients, as early diagnosis and treatment will decrease the overall incidence of morbidity and mortality. This case demonstrates a rare presentation of Graves disease in the ED in a young patient with thyroid storm as the cause of her first afebrile seizure.

## CASE REPORT

A female aged two years and 11 months was brought to the ED by ambulance for a generalized tonic-clonic first-time seizure. The patient had otherwise been in her usual state of health with no recent febrile illnesses. Her mother noted that the patient had an acute episode of unresponsiveness with a blank stare and called 9–1–1. Upon emergency medical services arrival, the child developed generalized tonic-clonic seizure activity. She received a weight-based dose of 1.7 milligrams (mg) of midazolam en route to the hospital, which resolved the movements. Upon arrival to the hospital, the child was no longer actively seizing and appeared to be sleepy, consistent with a postictal state. The estimated total seizure length was approximately two minutes. The patient had no notable past medical history. Family history was notable for Graves disease in the patient’s mother.

**Image 1.**
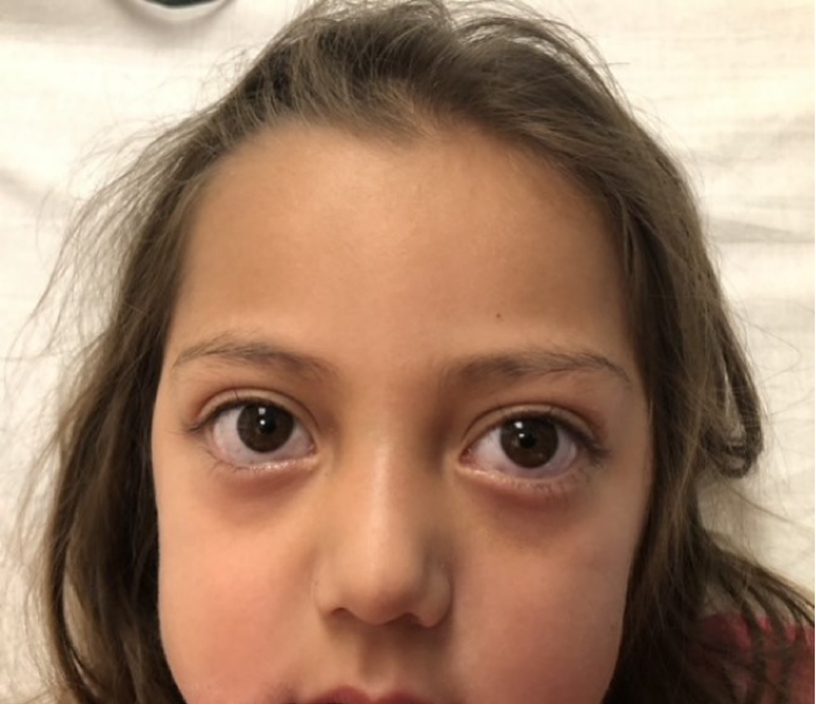
Exophthalmos noted in a pediatric patient with Graves disease.

**Image 2.**
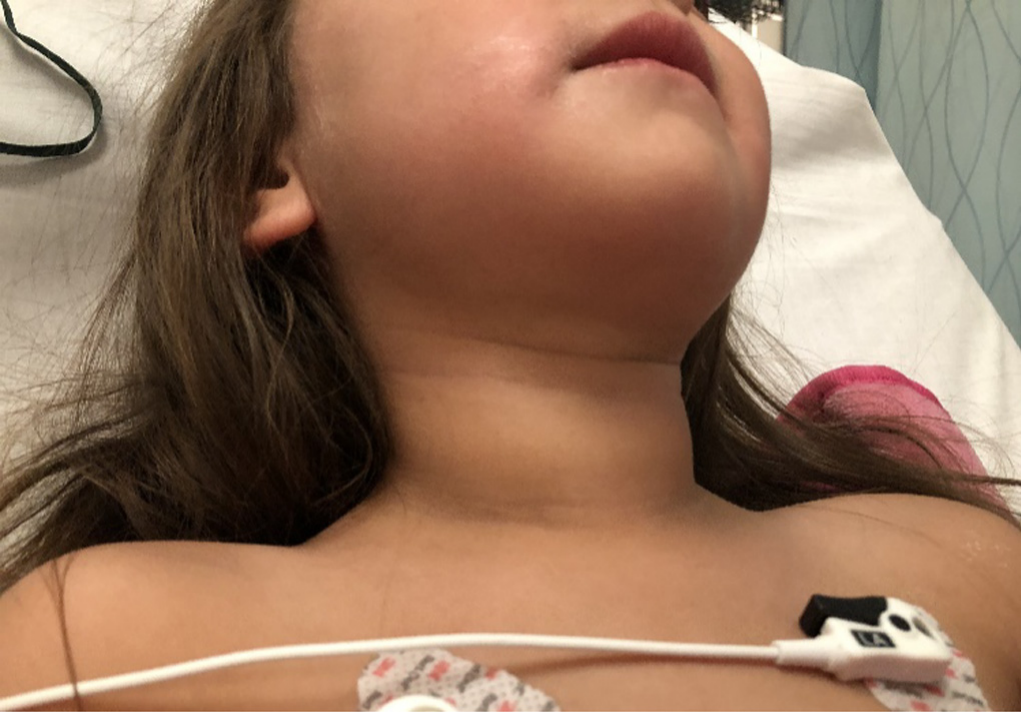
Palpable goiter noted in a pediatric patient with Graves disease.

Vital signs on arrival were as follows: temperature 37.9° Celsius measured rectally; heart rate 188 beats per minute; respiratory rate 27 breaths per minute; and blood pressure 125/62 millimeters of mercury. Her height was 103 centimeters, 99^th^ percentile, and her weight was 16 kilograms, 87^th^ percentile. Her physical exam was notable for exophthalmos (Image 1) and small goiter (Image 2) but was otherwise unremarkable, including a non-focal neurological examination. Of note, her tachycardia and widened pulse pressure persisted after resolution of her seizure and postictal period.

Her electrocardiogram was notable for left ventricular hypertrophy and sinus tachycardia but had no evidence of ischemia or abnormal intervals. Her laboratory evaluation was notable for a normal fingerstick blood glucose, elevated alkaline phosphatase with mild transaminitis, mild microcytic anemia, undetectably low thyroid-stimulating hormone (TSH) and unmeasurably high free thyroxine.

The pediatric endocrinologist was consulted who, based on the patient’s weight, recommended starting atenolol 12.5 mg, methimazole 7.5 mg, and 1 milliliter (mL) potassium iodide-iodine (Lugol’s saturated solution of potassium iodide {SSKI}) solution (to be given one hour after atenolol and methimazole). After treatment was initiated, the patient’s heart rate improved to the 160s, and she was admitted to the pediatric intensive care unit (PICU). While in the PICU, further levels were obtained and were notable for unmeasurably high free triiodothyronine (T3), unmeasurably high total T3, and elevated thyroid-stimulating immunoglobulins (328% baseline). While in the PICU, the patient was continued on atenolol, methimazole, Lugol’s solution, and hydrocortisone 50 mg every eight hours. She was in the PICU for approximately 24 hours and then transferred to the medical ward.

The patient remained in the hospital for one week and was discharged home with methimazole, propranolol, and SSKI. She was followed in endocrinology clinic multiple times over the next few months. The SSKI was eventually stopped, and propranolol was changed to atenolol. She had trouble with compliance with medications as the formulations available for atenolol are not child friendly. She was referred to another hospital system for a total thyroidectomy given inability to tolerate swallowing pills. She has not had recurrent seizure activity since initiating thyroid treatment.

## DISCUSSION

Graves disease is uncommon in young children, and seizure is a rare presentation of thyrotoxicosis secondary to this disease. Hyperthyroidism is the state of having higher than normal levels of circulating thyroid hormone; this disorder can present sub-clinically or profoundly symptomatic. Symptomatic patients are commonly considered to have thyrotoxicosis, although the terms thyrotoxicosis and hyperthyroidism are sometimes used interchangeably.^8^ Hyperthyroidism/thyrotoxicosis has a case rate of 1–14 cases/100,000 patient-years in children under 17, with Graves disease being the most common cause.^9^ ^–^ ^12^ Graves disease also appears to be increasing in incidence when compared to the last 20–30 years and affects females more frequently than males in a 3–4∶1 ratio.^9^ ^,^ ^11^ Graves disease is the result of TSH receptor stimulation by thyrotropin receptor antibodies causing increased thyroid hormone production and release.^2^ ^,^ ^10^ ^–^ ^13^

While Graves disease is the most common cause of thyroid storm, diagnosing thyroid storm itself can be difficult.^5^ ^,^ ^6^ Thyroid storm, the extreme manifestation of thyrotoxicosis, is a life-threatening condition that results in multiorgan dysfunction.^5^ ^–^ ^7^ ^,^ ^13^ All systems can be affected, and patients commonly demonstrate some combination of the following: central nervous system (CNS) alteration; cardiac manifestations including tachycardia, other arrhythmias, and sometimes congestive heart failure and cardiovascular collapse; and gastrointestinal symptoms such as vomiting, diarrhea, and hepatic failure.^5^ ^,^ ^6^ ^,^ ^11^ There are no universally accepted criteria or validated clinical tools to confirm the diagnosis of thyroid storm. Two decision tools used in adults are the Burch-Wartofsky point scale (BWPS)^14^ and the Japanese Thyroid Association (JTA)^15^ diagnostic criteria. The BWPS, which was developed in 1993, assigns points based on level of organ dysfunction with scores >45 suggestive of thyroid storm, between 25–45 suggestive of impending storm, and scores <25 unlikely to be thyroid storm. The JTA, developed in 2012, requires thyrotoxicosis to be present (the BWPS does not incorporate thyroid studies) and evaluates for the presence of organ dysfunction. The JTA suggests thyroid storm based on a combination of systems involved rather than the severity of symptoms. Notably, seizures are included in the BWPS but not in the JTA. Given the rarity of pediatric thyroid storm, these decision tools were developed in adult populations and their extrapolation to pediatric populations must be done with caution.

Thyroid storm can affect the CNS in several ways including restlessness, agitation, lethargy, delirium, psychosis, seizures, and coma.^5^ ^,^ ^6^ ^,^ ^14^ However, it is worth noting that seizures in the setting of thyroid storm are relatively uncommon; this is likely why seizure is not included in the JTA diagnostic criteria.^10^ ^,^ ^13^ Some animal studies suggest that thyroid hormone can lower the seizure threshold.^13^ Thyroid storm is a well-known cause for developing hyperthermia, which leads to a decreased seizure threshold in children. Given that the seizure threshold for children is already lower than that for adults, these factors likely further exacerbate the risk of seizing. Pediatric patients may be more likely to have seizures in the setting of thyroid storm.^10^

## CONCLUSION

Thyroid storm is an uncommon illness with high mortality; thus, emergency physicians must make this diagnosis and initiate treatment promptly. Thyroid storm should be considered in an altered and tachycardic patient and should be considered in the differential diagnosis for a child presenting with seizure. Early diagnosis and treatment reduce the incidence of morbidity and mortality. A detailed physical exam evaluating for exophthalmos and goiter, family history, and careful attention to vital sign abnormalities that persist after resolution of the seizure can facilitate making this critical diagnosis.
